# Explaining opinion polarisation with opinion copulas

**DOI:** 10.1371/journal.pone.0183277

**Published:** 2017-08-22

**Authors:** Nikolaos Askitas

**Affiliations:** IZA – Institute of Labor Economics, Schaumburg-Lippe-Str. 5/9, D-53113, Bonn Germany; University of Oxford, UNITED KINGDOM

## Abstract

An empirically founded and widely established driving force in opinion dynamics is homophily i.e. the tendency of “birds of a feather” to “flock together”. The closer our opinions are the more likely it is that we will interact and converge. Models using these assumptions are called bounded confidence models (BCM) as they assume a tolerance threshold after which interaction is unlikely. They are known to produce one or more clusters, depending on the size of the bound, with more than one cluster being possible only in the deterministic case. Introducing noise, as is likely to happen in a stochastic world, causes BCM to produce consensus which leaves us with the open problem of explaining the emergence and sustainance of opinion clusters and polarisation. We investigate the role of heterogeneous priors in opinion formation, introduce the concept of opinion copulas, argue that it is well supported by findings in Social Psychology and use it to show that the stochastic BCM does indeed produce opinion clustering without the need for extra assumptions.

## 1 Introduction

Opinions are important in economics just as they are in social sciences in general. Bubbles, manias and information cascades form in opinion spaces about expectations and they evolve in a self-organising manner. They are the outgrowth of adaptive dynamics whose understanding is of pressing significance. Clustering and polarisation, even without artificial communication obstacles, appear to persist and abound even as we are able to communicate information to each other without geographic, temporal or medial obstacles. In fact it is, perhaps paradoxically perhaps not, in this time of ease of communication that we are witnessing a pronounced misalignment of perception and fact (http://perils.ipsos.com/) or such utterly illogical phenomena as “post-truth politics”. We are thus reminded of the importance of social psychology in opinion formation.

The appearance of the first speculative bubbles coincides with the advent of the newspapers ([1] (p. 101)), the volatility of the stock market in the 1920s with the proliferation of the telephone, the stock market boom of the 1990 and the subsequent collapse of the new economy is related to the widespread adoption of the internet, and the emergence of social media after 2000 lead us to the collapse of 2008 ([1] pp. 181-182). The more enhanced the capacity for interpersonal communication becomes the more accelerated the “contagion of ideas” and the more prominent their role in the formation of speculative bubbles. The World Economic Forum in its 2013 report identified “digital wildfires in a hyperconnected world” as one of the major global economic risks. Which, if any, aspects of our internet-centred society might help explain the observed opinion cleavages given that there is no longer an exogenous structure (e.g. distance or access) obstructing or limiting the exchange of ideas?

Besides decreasing the diameter of the graph of connections among economic agents, technological innovation increases the ability to communicate on more than one topics at the same time and this is what this paper is about. It discusses opinions dynamics in the presence of more than one “topics” or “issues” and gives shape to the thought that whether we will agree with each other on a given topic is certainly not completely immune to our differences on other issues, a fact which is well documented in Social Psychology. Our opinions are influenced by the context in which we formulate them ([2]) and are some times influenced by what we erroneously think others think thus often producing unexpected aggregate effects (e.g. pluralistic ignorance). So it is natural to conjecture that the more opinions we reveal to each other the more likely it becomes to get cross-topic contamination or to reveal an opinion gap too large to bridge.

In Social Media where [1] proposes that the contagion of ideas now takes place we are more than just our opinion on whether stock or housing prices will go up. We reveal just about everything to the other making issue spill-over possible and its study relevant. We propose therefore that while in the hyperconnected world the diameter of the information graph decreases, the distances in opinion space increases in tandem with the fact that we turn agents from opinion scalars to opinion vectors. We highlight the role of heterogeneous priors in opinion dynamics and show how dimensionality matters and in fact appears to be an elusive confounding factor which might help explain the persistence of opinion cleavages.

By using the idea of opinion copulas (which formalizes topic spill-over and topic contagion i.e. covariance of opinions on different topics) I show theoretically, by example, analytically and by simulation that in a world of uniform, univariate opinion distributions, polarisation arises if agents reveal, care about and are able to track more than one topics at a time something which becomes more likely in the context of Social Media and an increasingly algorithm-driven society.

This paper builds on a large literature strand in statistical physics and network science ([3]) and is closest to the small strand of multidimensional opinion dynamics therein ([4]). It compares however best in scope and approach but also in its attempt to bring the physics literature closer to theoretical economics and mathematical sociology with [5], where misinformation cascades are discussed, [6], where confirmation bias and social influence are modelled by a variant of the bounded confidence model and to [7] where in addition to a social “integration force” which leads individuals to move to the weighted average of like minded peers, a Durkheimian “individualisation force” is modelled as random, zero-mean noise with variance proportional to the density of close-by peers. This paper is particularly related to the latter two papers as it shares with them an attempt to explain polarisation in light of the fact that the stochastic BCM fails to do so. Where this papers differs is that it does not need to modify the BCM model but to highlight the role of opinion correlation and dimensionality. In a more general sense it is also related to the strand of literature on the interface of computer science and economics in the sense of [8]. Finally, conceptually this paper is closer to [9]. The Schelling model is an adaptive model where agents, who are assigned a binary attribute (meant to model skin color), move on a line (which models a residential area) because they have a mild preference for the density of their own binary attribute in a neighbourhood of their placement. By thinking of the binary attribute as a prior “opinion” which is already polarised and the placement on the line as as continuous opinion which is influenced by the binary prior we only need make a small modification and we can recast the Schelling model into a special case of an opinion model with a heterogeneous polarised prior.

## 2 Method

### 2.1 The role of heterogeneous priors in opinion formation

In discrete time opinion models one fixes a single topic of interest and then one updates opinions of agents on that topic using a function (homophily) which takes into account the opinions of agents on that same topic in prior rounds. I call these homogeneous priors. In the presence of two or more topics the possibility arises that the opinions of agents on each of these topics in subsequent rounds is influenced not just by the opinions of agents on the respective topic (which would be the case of homogeneous priors) but also by the opinions on some of the other topics. I call these heterogeneous priors.

In the expression “birds of a feather flock together” there are two variables. One is the *type of the feather* (the heterogeneous prior) and the other is position in space (towards which to flock), which would be the univariate opinion. The idea behind [9] is exactly to investigate how a mild preference for like feathered others (skin color) leads us to flock together and segregate. Paradoxically nowhere in the literature on opinion dynamics do heterogeneous priors play a role even though there is a large amount of evidence from social psychology, from as far back as in the fifties ([2]), that individuals’ opinions may well be impacted upon by factors exogenous to the issue at hand. Opinion dynamics have been studied mostly as if they were about real bird flocking as in [10].

In reality we hold opinions on a variety of issues and influence each other while we form them. Some times we hold an opinion for reasons not intrinsic to ourselves but which depend on which views we think others might hold or not. The bounded confidence model (BCM) assumes that we interact with opinions in a certain proximity to ours (bounded confidence) and that we then update our opinion by moving closer to each other (homophily). So opinion formation is an adaptive, self-organising process with a high degree of endogeneity. The BCM models opinion as a value in the unit interval (like a percent) and opinion distance is computed on the same single issue. This means that one needs to assume initial values with usually a uniform distribution. As time evolves the homogeneous priors (i.e. the priors in a single, one dimensional opinion space) determine proximity and hence interaction. Given what we know about the susceptibility of opinion formation to social psychological influences this appears to be a somewhat restrictive view however and passes on an opportunity to ask a core question: How do individuals form an opinion in the first place? In other words where do the initial values, the BCM assumes, come from? We might start nearing an answer by stating the obvious: opinions are held on “issues” and “topics”. Hence the real question is: where do issues come from? Before we can hold an opinion on the merits of a topic it needs to become an issue i.e. gain in salience. In other words it needs to go from identically zero salience (issue does not exist) to having some kind of non-zero values. In fact the degree of salience we assign to a topic, whether or not we think it is an important one, is the first type of opinion we hold on it.

So the current literature cannot possibly deal with topic salience as an opinion, until after it enters the public sphere, because the initial values of topic salience are born from heterogeneous priors. When homophily has no homogeneous priors on a given topic, to work with, and we are faced with the need to formulate an opinion on that topic, we can only look at priors on other topics which reality exposes us to. This is for example the case when we are more likely to disagree with someone on a topic if we feel antipathy for them (a feeling which is based on totally different factors exogenous to the topic at hand). Whether or not we agree with a *new* monetary policy of the European Central Bank has to do with our own “intrinsic” convictions (if such things exist) but is also influenced by whether our own Finance minister thinks highly of it or not. We come to think that migration policy or the refugee crisis are important and relevant topics or not because politicians we trust or don’t trust say they are worthy of our attention. Those same politicians (their other priors) and our social environment, our peers, influence our opinion on whether to allow more migration or take in more refugees. In the absence of homogeneous initial values heterogeneous priors are the only thing we have to go by.

The initial values the BCM assumes come up in a world with a prior structure of topic variety, topic salience and distributions of heterogeneous opinions. In other words when we consider homophily in a given, fixed, univariate, opinion space we have skipped the important step of considering homophily in the space of priors on other topics i.e. heterogeneous priors. Before we make up our mind on whether the refugee problem is an important one or whether a travel ban on citizens of certain countries is a relevant and important thing to consider we might want to know who else says it is and what type of proximity we have with these other people in a number of socioeconomic contexts that are revealed to us. Are they political figures that we agree with? Are they of the same race? What other opinions do they hold and how do they compare with ours?

As we go through life there are many contexts where different priors might be revealed to us to a different extend in tandem with a single emerging topic. The people at our church, our coworkers, the newspapers we read, our neighbourhood, our Facebook friends, our Twitter followers, the Twitter accounts we follow, a radio talk show host, an online support group, our boss, our employees, are all examples of innumerably many contexts in which different types, numbers and combinations of priors are revealed to us which help shape the initial value of our opinion on a new topic. But as soon as we think of these different possibilities of revealed priors we talk about vectors of opinions hence opinion copulas.

**Fig 1:**
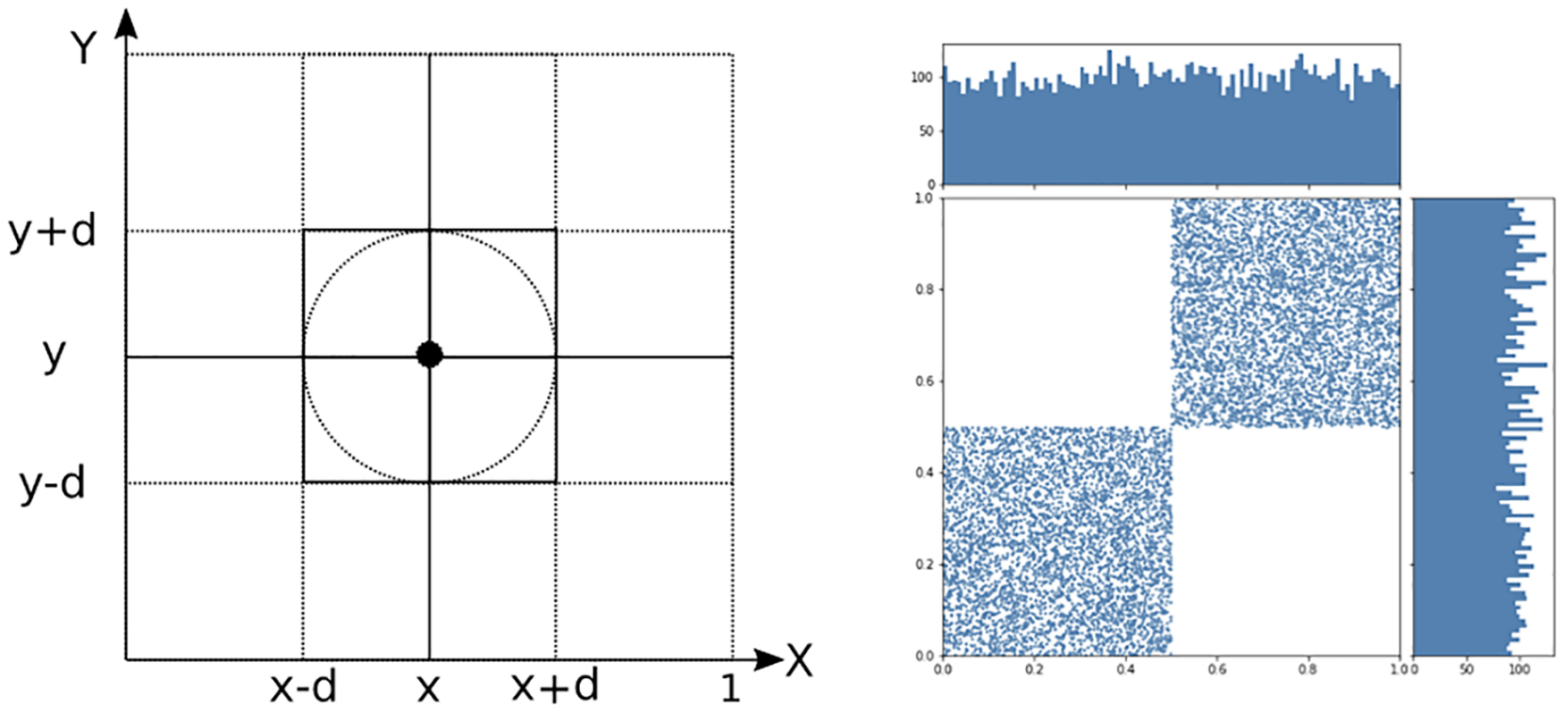
Left: Probability of interaction equals volume of neighbourhood and drops as dimension increases. For fixed bound of confidence *d* the probability of interaction drops with the number of variables observed. Right: 2-dimensional copula of uniform marginals which happens to be an ordinal sum. 10000 opinion agents with two opinions. Both marginal opinions are uniformly distributed but the opinion copula produces a pronounced cleavage which will form two clusters according to Eq 3. The mild requirement that opinion agents mostly preserve their side of the center leads to polarisation if distance is computed using both opinions.

Obviously if the priors we are exposed to are already polarised, homophily along these priors will polarise the newly arisen topic as well. For example if it is the right wing who propose not to take in any refugees we might find it hard to hold a matter-of-fact, nuanced position on how many refugees to take in if our politics are left and we might thus risk being put in the company of right wingers. These considerations might also partially explain such polarisations as pro-life vs. pro-choice or even irrationalities such as global warming denialism. But where do the prior polarisations come from? Can polarisation be born in a world of uniform univariate distributions? We show it is indeed possible to produce polarisation on a new topic by means of the stochastic BCM using heterogenous priors none of which is polarised. The answer comes by means of opinion copulas since it is possible, for example, to get two dimensional agglomerations from uniform univariate marginal distributions (as in, for example, Fig 1 right hand side). The novelty of our paper is not so much to consider vector valued opinions as there is literature on this type of opinions already (see for example the literature review in [11]) but rather to point out the difference between computing univariate vs multivariate distance and to use copulas to demonstrate that heterogeneous priors can be seen as uncovered hidden confounding factors.

### 2.2 Opinion copulas

If *d* is the bound of confidence then in a context where only one opinion is revealed or taken into account the range of social influence around the agent is an interval of length 2*d* whereas in a context where *n* opinions are observed and might be acted upon the situation becomes a bit more complex. Assume, for simplicity, that the intervals around each marginal opinion of radius *d* are within the unit interval. Then, for each of the marginals, the probability that another random agent is within the bound is 2*d* while, the probability for this to happen for all marginals simultaneously is give by the volume of an *n*-dimensional ball of radius *d* in some normed metric. We know that the volume of an *n*-dimensional ball of fixed radius tends to zero as *n* goes to infinity (see [12]). This means holding the bound of confidence fixed the radius of influence around agents tends to zero as dimension goes to infinity. A moments thought will convince the reader that this holds true even if we relax the bound as *n* grows in any kind of reasonable way. This has an intuitive and sound interpretation in reality: the more issues we take into account the harder it is to agree with each other.

We reveal different combination of opinions in different contexts. When we do so it is reasonable to assume that in computing our opinion distance from the other we take all of these opinions into account. These might include the opinion we are updating or not. For example when the opinion we are focusing on has no initial values as is the case when a topic acquires its salience then the bundle of opinions might vary but will not include the opinion in question or its degree of salience. We will call such opinion combinations and their joint distribution of agents along these opinions **opinion copulas** for obvious reasons. In Fig 1 (right hand side) we see such a 2-copula of opinions. Notice that both marginals are uniform and that the 2-dimensional distribution is the *ordinal sum* of two uniform distributions with respect to a splitting of the unit interval in the middle (see [13]). This copula of opinions appears to be clustered already and its dynamics can be seen to lead to a bimodal distribution for the resulting marginals. This copula of opinions appears to be an artificially chosen one but it is in fact the idealised version of an opinion copula both quite general and quite likely. It is the result of the statements “agents vary their different opinions freely but tend to keep on the same side of the center across issues”. Applying Eq 3 we see that this will surely lead to segregation in the deterministic model and also in the stochastic BCM as we will see in Section 4.

## 3 Model

In the standard bounded confidence model of opinion dynamics among a population of *N* agents, an agent *i* holds an opinion *x*_*i*_(*t*) ∈ [0, 1] at time *t* which she updates by means of the rule:

$$\begin{array}{c} \begin{array}{c} x_{i}\left(t+1\right)=x_{i}\left(t\right)+\mu \left(x_{j}\left(t\right)-x_{i}\left(t\right)\right)+\xi_{i},\;\text{if}\;|x_{j}\left(t\right)-x_{i}\left(t\right)+\zeta_{i}|<d, \end{array} \end{array}$$ (1)

where *μ* < 1 is the **convergence parameter**, *d* is a fixed **bound of confidence** and $\xi_{i},\zeta_{i}\in N\left(0,\sigma_{i}^{2}\right)$ is noise at the calculation of the new opinion and the calculation of proximity respectively. When *ξ*_*i*_ = *ζ*_*i*_ = 0 we have the deterministic BC model. The inequality in Eq (1) will be called the **proximity condition**. In each round two randomly chosen agents compute their distance and update their opinions. A differential equation is known to hold for the density function *ρ* of the opinions of the deterministic model which is similar to Fick’s second law of diffusion:

$$\begin{array}{c} \begin{array}{c} \frac{\partial \rho }{\partial t}=\frac{d^{3}}{2}\mu \left(\mu -1\right)\frac{\partial^{2}\rho^{2}}{\partial x^{2}}. \end{array} \end{array}$$ (2)

Equation 2 is derived in [14] by essentially the same argument as in [15]. It shows that peaks of agent density at opinion values (i.e. points of *x* where $\frac{\partial \rho }{\partial x}=0$ and $\frac{\partial^{2}\rho^{2}}{\partial x^{2}}<0$) have their agent density increase in time (because 0 < *μ* < 1).

It is well known that smaller values of *d* produce more clusters but that the introduction of a little bit of noise makes them all collapse to a single cluster i.e. it produces consensus.

We consider the higher dimensional analogue of the BCM and will be interested in comparing what happens when more than one opinions update based on the distance computed from their own priors versus when they update using the vector distance of all of their values. In (S1 Infinitesimal Opinion Dynamics for Opinion Copulas) we show that the density *ρ* of each marginal opinion in the deterministic *n*-dimensional BCM obeys:

$$\begin{array}{c} \begin{array}{c} \frac{\partial \rho }{\partial t}=\frac{{\left(2d\right)}^{n+2}}{16}\mu \left(\mu -1\right)\nabla^{2}\left(\rho^{2}\right), \end{array} \end{array}$$ (3)

where $\nabla^{2}=\sum_{i=1}^{n}\frac{\partial^{2}}{\partial x_{i}^{2}}$ is the Laplace operator.

Eq 3 implies that, as in the one dimensional case, in the *n*-dimensional deterministic BCM density peaks become more pronounced and valleys diminish. Moreover there is a role to be played by the dimensionality of the copula: if *d* < .5 then density remains unchanged in time as *n* goes to infinity. It is of course unrealistic to expect that an infinite or even an ever higher number of opinions will play a role since agents are limited. However besides it being an instructive fact there are scenarios in which realistic variants are indeed possible. For example imagine a case where agents randomly interact computing distances based on randomly joint couples of opinions drawn from a higher dimensional space. On Facebook I might compute our distance using the political colouring of your likes together with your opinion on the travel ban for Muslims but on the varsity soccer team I might take into account your humour and your athletic vehemence.

So the core experiment is to investigate what happens if we take two or more opinions related with a random covariance matrix, run the stochastic BCM by computing proximity using the multivariate distance and see whether or not we obtain clusters and whether or not they persist longer than in the univariate case.

## 4 Results

**Fig 2:**
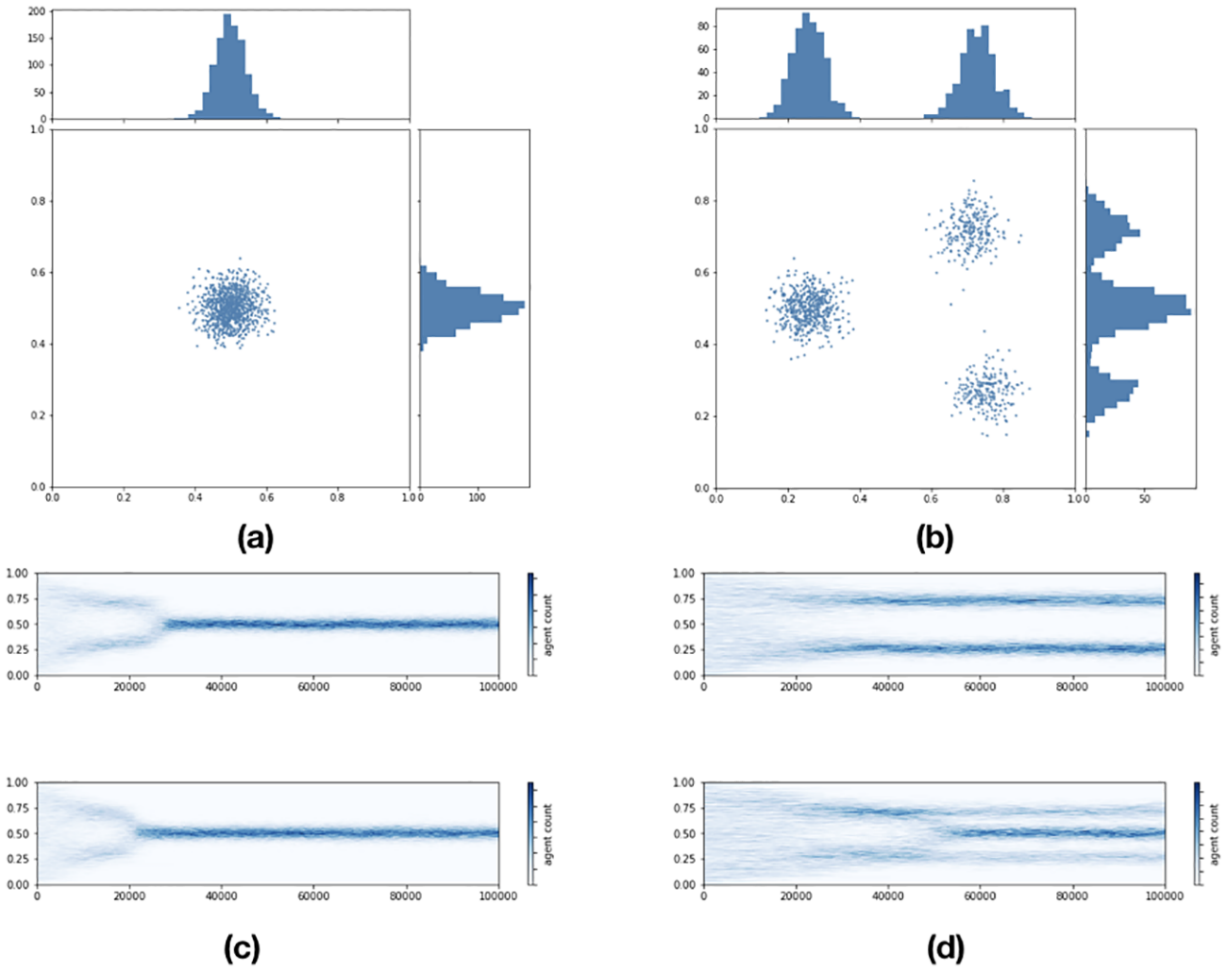
One thousand opinion agents, 2 topics, 100000 iterations, *d* = .26, noise from N(0,.03), *μ* = .4—(a) Independent evolution. Final state, (b) Evolution in tandem. Final state, (c) Independent evolution. Time distribution, (d) Evolution in tandem. Time distribution.

**Fig 3:**
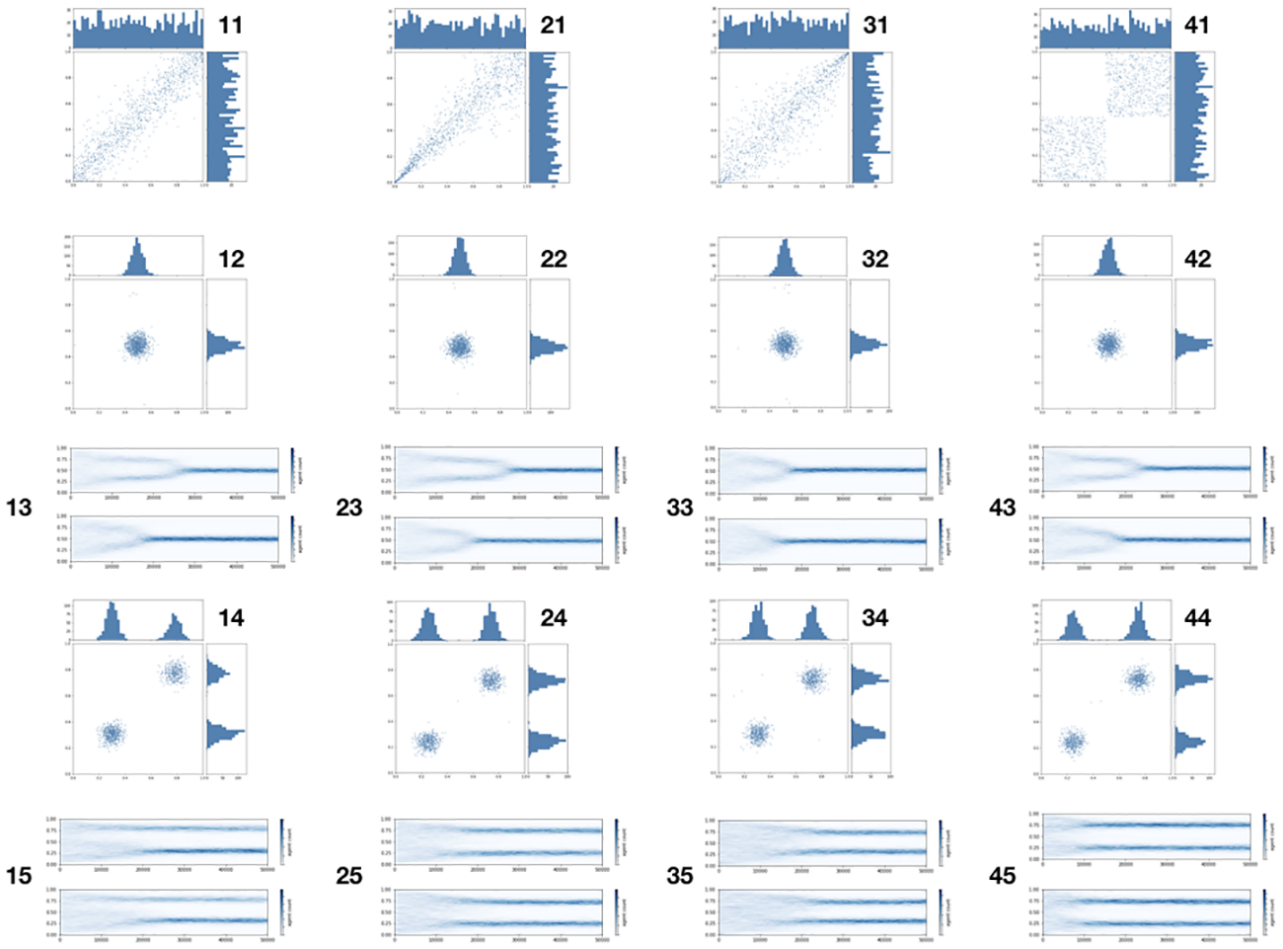
1000 agents, four two-topic copulas, 50000 iterations, *μ* = .4, *d* = .26, noise from N(0,.03)—(11) Frank, (21) Clayton, (31) Gumbel, (41) Ordinal sum. (12, 22, 32, 42) Final state: uni- variate, (13, 23, 33, 43) Marginals in time: univariate, (14, 24, 34, 44) Final state: bivariate, (15, 25, 35, 45) Marginals in time: bivariate.

In this section I run several simulations. The first class of simulations involves the stochastic BCM with univariate and multivariate distance condition. I do this for a random copula of univariate marginals (Fig 2) and for four different well known copulas (Fig 3): the three best known Archimedean copulas of Frank, Clayton and Gumbel which occur in finance and the ordinal sum of univariate copulas around the middle which has a nice real world interpretation. In all cases I show that the bivariate evolution leads to clusters long after the univariate clusters have collapsed to consensus. The second type of simulation involves the Schelling model. This is basically the same as in [9] except that we allow piling and interpret the unit interval not as geographical location but as the range of a continuum opinion variable and the binary attribute is a fixed opinion prior.

I ran several thousand rounds of the two dimensional BCM with 1000 agents computing opinion distance in a univariate manner and in a bivariate manner plotting two dimensional histograms of the time evolution of the marginal and as well as the initial and final state of the system. Noise is picked from N(0,σ) for some *σ*. I report the values of *μ* and *d*. Fig 2 summarizes the results.

The top left and top right shows the final state of the distribution of agents in the cases of univariate distance and bivariate distance respectively. The graphs depict the scatter plot of agents along the two opinion axes while attached on the top and right hand side of each one of them we have histograms of the marginal opinion distributions. The initial values are the same in both cases and are randomly chosen from uniform distributions put together into a copula with a random covariance matrix. Below each of these two graphs we have 2 dimensional histograms of each marginal opinion. We see that multimodal distributions of the marginal opinions in the bivariate BCM persist long after the univariate model produced consensus.

Observing the multimodal distributions of the marginals in the final state on the top right hand side of Fig 2 we see that if we did not know that agents compute their distance using a second opinion as well we would have a conundrum which would have us scratching our heads in a manner similar to when the BCM fails to explain the prevalence of cleavages everywhere. By taking into account the second variable we uncover a hidden confounding factor. In a hyperconnected world it is possible to have multiple successive copulas in effect accounting for random appearance of clusters with opinion agents being dynamically reallocated in opinion space time.

I do the same simulation for the three best known Archimedean copulas of Frank, Clayton and Gumbel, (columns one, two and three of Fig 3) as well as for the ordinal sum of two univariate marginals with the same robust results as predicted by Eq 3. The Archimedean copulas form the initial conditions of the experiment in this case.

The ordinal sum in Fig 3 is a very interesting case because it says that “while opinion agents are otherwise free to choose an opinion, they come down on the same side of the center across issues”. One can obviously extend this to more than two ordinal summands. For example the ordinal sum of univariate copulas for the partition {.45, .55} of the unit interval may be interpreted as saying that people choose randomly but the left of center stay left the right of center stay right and the center people move about the middle but stay within [.45, .55]. This case would polarise as well if the copula of opinions was used to compute opinion distance. Finally while these are idealised ordinal sums obviously the results hold for noisy versions of “sums” as well as in “while opinion agents are otherwise free to choose an opinion they mostly come down on the same side of the center across issues”.

Ordinal sum opinion copulas of this kind might well occur due to intrinsic agent properties, because, for example, people might have left or right disposition. The bivariate copula then produces clusters in two dimensional space which, in informational environments where agents reveal and evaluate both opinions, agent interaction lead to the clusters in two dimensional space getting so pronounced that the new marginals become bimodal as well.

Finally I run essentially the Schelling model interpreting the position on the unit interval as opinion by allowing continuous values.

**Fig 4:**
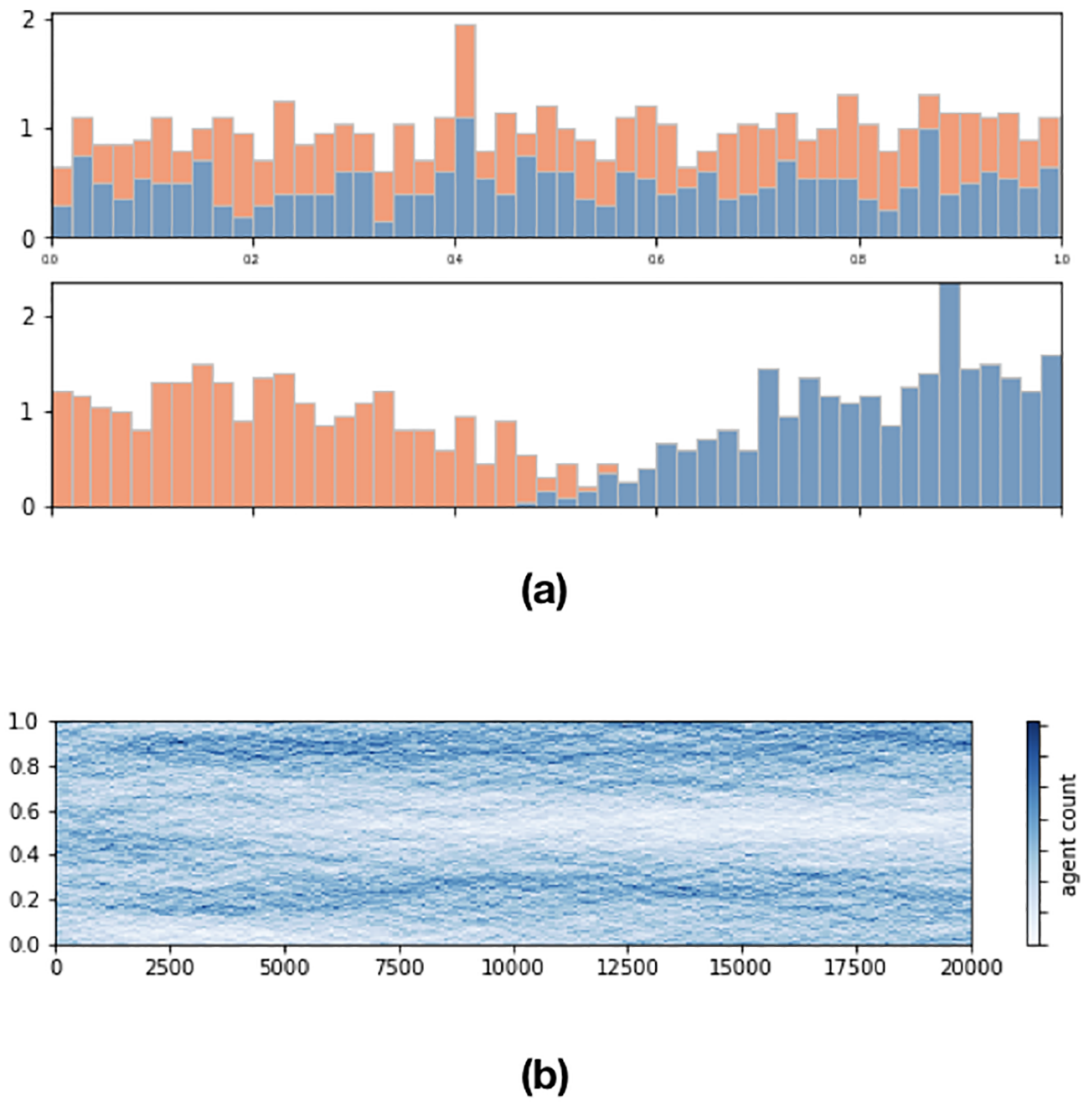
One thousand opinion agents, 20000 iterations, *μ* = .2, *d* = .4, tolerance .5, noise from N(0,.001). (a) Stacked histograms of uniformly distributed agents (top) in continuous opinion space with a fairly assigned, heterogeneous binary “color” attribute, preferring not to be a minority “color” in a neighbourhood of their opinion arrange themselves in cleavages with two modes (bottom), (b) Two dimensional histogram of evolution in time.

Each agent has a “color” attribute and a continuous opinion which he updates as follows. For some radius *d* around him the agent computes the average color and if the other color prevails he seeks to alleviate that by moving left or right by a step of *μ* if that improves the color discrepancy. Schelling’s “colors” are interpreted as a polarised binary prior opinion which for the sake of simplicity is kept unchanged. Obviously it can also be an attribute rather than an opinion like religion, party affiliation or race. The results are summarised in Fig 4. As in [9] the simulation shows that a preference not to be in the “color” minority in a neighbourhood of one’s opinion leads to color-wise homogeneous opinion equilibria which (and this is new in this variation) are multimodal. The number of modes increases as the size of the range (i.e. *d*) decreases and the cleavages are more pronounced when *μ* is larger. In Fig 4 there are two modes for *d* = .4. Obviously the two mode equilibrium remains an equilibrium for smaller values of *d* even though the update algorithm may not reach it. In other words, for smaller values of *d*, assuming a two mode equilibrium as our initial condition our update algorithm will preserve it but starting from random initial conditions might not yield it. This variation of the Schelling model appears suitable to explain why major divides such as Republican vs Democrat appear to polarise other topics as well such as pro-life vs pro-choice etc.

Last but not least of interest is also to combine the Schelling model and the BCM. It is entirely possible that opinion agents care both about a prior in the sense of not belonging to a minority with their continuous opinion but also apply homophily merging with those that are closer to them. Obviously the cleavages from the Schelling model will lead to clusters with the BCM according to Eq 3.

## 5 Discussion

Investors with a bearish or bullish outlook, the governing council of the European Central Bank voting on a quantitative easing program, company employees forming an opinion about their boss, voters considering how to cast their vote or youth considering how to wear their hair. We hold opinions about issues and each other about the future and the past about financial outlooks, fashion, art and anything else. We some times stick to our views and sometimes let others influence what we think about someone or something. We might feel better when we are among like-minded peers and we may some times adopt or reject an opinion based on who else is holding it, discriminating on the basis of traits more or less relevant to the issue at hand, feeling more or less comfortable with an opinion we hold depending on whether we like the company of our like-minded peers.

Individuals enter the process of opinion formation with a possibly subconscious optimisation problem of finding a compromise between their intrinsic opinion and conforming with others, in a possibly noisy environment, stimulated to iteratively update their stance at varying numbers or frequencies depending on various individual or societal parameters. The process is highly endogenous and its dynamics may or may not be able to reach a theoretical equilibrium even when one exists.

Finally whether a topic will elevate from obscurity and become an issue in other words whether it will acquire enough salience to make us feel compelled to have an opinion is a process which can only take place using heterogeneous priors: in forming an opinion about the importance of an issue we have no initial values to go by and we are left with using heterogeneous opinions.

We introduced the concept of opinion copulas and extended the BCM model to multivariate opinion proximity and showed that dimensionality matters and that it deserves more investigation when we think about opinion dynamics. Opinion copulas are suitable for the modelling of social psychological aspects of opinion formation.

## Supporting information

S1 Infinitesimal Opinion Dynamics for Opinion Copulas Infinitesimal opinion dynamics for opinion copulas. (PDF)
