# Supplementary material for: Explaining opinion polarisation with opinion copulas
Source: PLoS One. 2017 Aug 22;12(8):e0183277. doi: 10.1371/journal.pone.0183277 (PMC5568744; doi:10.1371/journal.pone.0183277)
Supplement: S1 Infinitesimal Opinion Dynamics for Opinion Copulas — (PDF) [file pone.0183277.s001.pdf]

Supporting information

S1 – Infinitesimal Opinion Dynamics for Opinion Copulas
We prove the higher dimensional analogue of Eq 1 derived in [1] :

$$\frac{\partial \rho}{\partial t} = \frac{d^3}{2}\mu(\mu-1)\frac{\partial^2 \rho^2}{\partial x^2}. \tag{1}$$

We prove namely that if n opinions move together then:

$$\frac{\partial \rho}{\partial t} = \frac{(2d)^{n+2}}{16}\mu(\mu-1)\nabla^2(\rho^2), \tag{2}$$

where ρ is the density function in the n-dimensional opinion space and $\nabla^2 = \sum_{i=1}^{n}\frac{\partial^2}{\partial x_i^2}$ is the Laplace operator. To prove this equation we mimic the argument in [1] which is essentially the same as in [2]. First observe that the negative contributions to the density $\rho(x)$ at a point $x \in [0,1]^n$ is essentially the probability that x will interact with opinions in its proximity i.e.:

$$-\rho(x)\int\limits_{y\in B(x,d)}\rho(y)dy, \tag{3}$$

where $B(x,d)$ is the neighbourhood of x of radius d in the p-norm with $p=\infty$ (it simplifies calculations by turning the integration domains into cubes and we can easily apply Fubini's theorem on changing the order of integration) On the other hand the positive contribution to the density $\rho(x)$ at a point x is the probability that an agent at opinion y will interact with an agent at opinion z and his opinion will become x i.e.:

$$\int\limits_{y\in B(x,\mu d)} dy\cdot\rho(y)\int\limits_{z\in B(y,d)} dz\cdot\rho(z)P(y+\mu(z-y)=x), \tag{4}$$

where $P(y+\mu(z-y)=x)$, is the probability that $y+\mu(z-y)=x$. Now summing negative and positive contribution from 3 and 4 we obtain the total change of the density ρ at x:

$$-\rho(x)\int_{y\in B(x,d)}\rho(y)dy + \int\limits_{y\in B(x,\mu d)} dy\cdot\rho(y)\int\limits_{z\in B(y,d)} dz\cdot\rho(z)P(y+\mu(z-y)=x), \tag{5}$$

For y near x and z near y we apply the higher dimensional analogue of Taylor and keep up to second order terms:

$$\begin{aligned}\rho(y) &= \rho(x)+\nabla\rho(x)(y-x)+\frac{1}{2}(y-x)^T H(\rho(x))(y-x),\\ \rho(z) &= \rho(x)+\nabla\rho(x)(z-x)+\frac{1}{2}(z-x)^T H(\rho(x))(z-x),\end{aligned} \tag{6}$$

where $\nabla\rho(x)$ is the vector of first derivatives of ρ at x and $H(\rho(x))$ is the Hessian. By substituting 6 into 5 and observing that first order and mixed second order terms vanish by symmetry we obtain the final formula by means of elementary calculus.

References

1. Neau D. Révisions des croyances dans un système d'agents en interaction; 2000. Available from: http://www.lps.ens.fr/{~}weisbuch/rapneau.ps.

2. Einstein A. Zur Theorie der Brownschen Bewegung. Annalen der Physik. 1906;324(2):371–381. doi:10.1002/andp.19063240208.
